# Knowledge, Attitude, and Practice on Antibiotics and Its Resistance: A Two-Phase Mixed-Methods Online Study among Pakistani Community Pharmacists to Promote Rational Antibiotic Use

**DOI:** 10.3390/ijerph18031320

**Published:** 2021-02-01

**Authors:** Faiz Ullah Khan, Farman Ullah Khan, Khezar Hayat, Tawseef Ahmad, Amjad Khan, Jie Chang, Usman Rashid Malik, Zakir Khan, Krizzia Lambojon, Yu Fang

**Affiliations:** 1 Department of Pharmacy Administration and Clinical Pharmacy, School of Pharmacy, Xi’an Jiaotong University, Xi’an 710061, China; fkhan@bs.qau.edu.pk (F.U.K.); farmankhan@bs.qau.edu.pk (F.U.K.); khezar.hayat@uvas.edu.pk (K.H.); jiechang@xjtu.edu.cn (J.C.); usmanmalik_ucp@hotmail.com (U.R.M.); krizlambojon@gmail.com (K.L.); 2 Center for Drug Safety and Policy Research, Xi’an Jiaotong University, Xi’an 710061, China; 3 Shaanxi Center for Health Reform and Development Research, Xi’an 710061, China; 4 Research Institute for Drug Safety and Monitoring, Institute of Pharmaceutical Science and Technology, Western China Science & Technology Innovation Harbor, Xi’an 710061, China; 5 Department of Pharmacy, Abbottabad Campus, COMSATS University Islamabad, Abbottabad 22060, Pakistan; tausifsafi95@gmail.com; 6 Department of Pharmacy, Quaid-i-Azam University, Islamabad 45320, Pakistan; amjadkhan@qau.edu.pk (A.K.); zakirkhan300@gmail.com (Z.K.); 7 Department of Pharmacology, Institute of Health Sciences, Çukurova Universitesy, Adana 01330, Turkey

**Keywords:** antibiotics, antibiotic resistance, rational drug use, community pharmacist

## Abstract

Antibiotic resistance (ABR) is an emerging global threat to public health. Substantial evidence has indicated that community pharmacists (CPs) can play a critical role in managing the ever-increasing threat of antibiotic resistance. This study aimed to determine the knowledge, attitude, and practices of CPs (*n* = 180) towards antibiotics and antibiotic resistance as well as to improve the rational use of antibiotics. A two-phase mixed-methods (quantitative and qualitative) online study was conducted in Pakistan from August 2019 to March 2020 by using validated questionnaires and semi-structured interview data. Different statistical methods were used to tabulate the quantitative data, whereas inductive thematic analysis was conducted to categorize themes from the qualitative data and to draw conclusions. Approximately 64.4% of the CPs were male (mean: 29–33 years old). Overall, CPs had good knowledge of and were familiar with multidrug-resistant organisms and their roles in ABR (65.6%, median = 1, and IQR = 1), although their knowledge was poor in differentiating some antibiotic groups with their respective ABR patterns (31.1%, median = 1, and IQR = 1). Most CPs have a positive attitude towards antibiotics, with most (90.0%) identifying ABR as a critical issue in public health (median = 1 and IQR = 0). Overall, CPs’ practices towards antibiotics were somewhat acceptable, where they leaned towards educating patients about the rational use of antibiotics (52.8%, median = 1, and IQR = 1). The two main themes discovered (antibiotics and counseling of patients) were related to self-medication, while educational intervention is the main subtheme. ABR is multifactorial, with subthemes related to budget, time constraints, incompetent staff, the absence of CPs, the lack of training, and the enforcement of laws and regulations being the needs of the hour in Pakistan. Effective antibiotic stewardship programs, patient education, and awareness campaigns about antibiotics and ABR along with training of the CPs are important factors that have to be addressed in a timely manner.

## 1. Introduction

Globally, community pharmacies are integral to healthcare systems and play vital roles in medication-provision services [1,2]. In the last few decades, pharmacy practices have morphed towards putting more emphasis on ensuring patient safety by offering effective treatment at a low cost [3], with a shift from merely dispensing medicines to clinical activities in modern community pharmacy practices [4]. Similar to other countries, community pharmacies in Pakistan are deemed to be the most accessible facilities for healthcare services [5]. The private sector makes up the major part of the Pakistani healthcare system and is predominant in pharmacies set up throughout the country [5,6,7]. 

Therapy effectiveness solely depends on accurate dispensing of medicines, and qualified community pharmacists’ professional competency is vital.

Dispensing antibiotics without prescriptions compounded by the unavailability of CPs is a major issue in low- and middle-income countries (LMICs) [8]. The knowledge gaps in antibiotics and antibiotic resistance (ABR) and the lack of legal features in antibiotic dispensing lead to inappropriate antibiotic dispensing practices in LMICs [8,9,10]. Historically, infectious diseases have been a primary cause of mortality in the world. Nevertheless, following Fleming’s discovery of penicillin in the 20th century coupled with improved sanitation, there was remarkably reduced mortality and burden correlated with infectious diseases [11,12,13]. In recent years, there has been growing attention on antibiotics since the inappropriate use of these medicines has led to the development of bacterial resistance [14,15,16]. The World Health Organization (WHO) has declared antimicrobial resistance (AMR) a major global problem [17], which has become a growing concern in the community [14]. 

It was reported globally in 2019 that at least 700,000 people die each year due to drug-resistant diseases [18]. In the United States, more than 2.8 million antibiotic-resistant infections occur every year and over 35,000 people die as a result [19]. The outcomes of ABR in low-income countries (LICs) and LMICs are even more notable since a higher prevalence of microbial infections occurs in these countries [20]. The AMR at the community level had the greatest scope, as this is where the majority of antibiotics are prescribed [21,22,23]. The WHO global strategy for containment of antimicrobial resistance highlighted the need for appropriate use of antimicrobials as well as the significant roles of healthcare providers in educating patients on the rational use of antibiotics [24]. Hence, the engagement of suitable stakeholders such as CPs is of vital importance to support the antimicrobial campaign and implementation of antimicrobial stewardship programs in the community. 

A survey in Malaysia reported that approximately 90% of CPs often communicate with clinicians or prescribers whenever there is any uncertainty in the appropriateness of antibiotic prescriptions and nearly 70% of CPs often educate patients with regards to the rational use of antibiotics [25]. Meanwhile, in China, over 78% of CPs were involved in screening antimicrobial prescriptions, but their engagement in antimicrobial campaigns was not satisfactory [26]. Previous studies in Pakistan have investigated the antibiotic use, antimicrobial resistance, and antimicrobial stewardship program [27,28,29,30,31], but earlier studies have reported on the role of CPs as antibiotic stewards [32,33]. However, to our knowledge, no study assesses the knowledge, attitude, and practice of antibiotics and their resistance among community pharmacists to promote rational drug use in Pakistan. Thus, the main objective of this study is to evaluate the knowledge, attitude, and perception of CPs in Pakistan regarding the nonprescription dispensing of antibiotics and how to improve the rational use of antibiotics. 

## 2. Materials and Methods

### 2.1. Survey Implementation 

A two-phase mixed-methods study design was used, which consisted of (1) an online questionnaire (quantitative) cross-sectional study and (2) a detailed telephone semi-structured interview (qualitative). The CPs worked full or part-time and had a connection with the services at the community pharmacies included in the survey. The present study has two parts: A quantitative phase 1 followed by a qualitative study (phase 2). Ethical approval was obtained from Xi’an Jiaotong University, Health Science Center Biomedical Ethics Committee (reference number: 2019–1256), which complies with the Declaration of Helsinki.

### 2.2. Phase 1: Quantitative Study

#### 2.2.1. Study Design and Settings

The online platforms were used between August 2019 and March 2020. Per the 2017 census, Pakistan has a total population of approximately 208 million, with a 2.4% population growth [34]. There are more than 80,000 CPs, with a wide geographical diversity [6,35,36]. Traditionally, it was difficult to conduct a study of CPs by personal visit. However, an online approach was selected for data collection and approximately 76 million people in Pakistan have access to the internet, while 37 million people are active users of various social media platforms [37]. A nationwide online survey was conducted in the four provinces along with Azad, Jammu, and Kashmir (AJK) and Gilgit Baltistan. Only pharmacists engaged with the community pharmacy services, full-time or part-time, were approached. 

#### 2.2.2. Study Instruments 

An extensive literature review was conducted, while different scientific search engines were explored for relevant articles for the questionnaire design. A questionnaire was developed and adopted from a previous study [38]. After the initial survey framework, the content and face validity of the instrument were tested by experts in the pharmacy practice research comprising two academicians and one biostatistician. Changes were made as recommended by the experts for a better understanding of the questions by CPs. 

Finally, a 27-item questionnaire with four sections for the final survey (Supplementary File S1) was developed. Section one comprised questions related to the participant’s demographics information such as gender, age, education (graduation: B.Pharm and PharmD, or postgraduation: MPhil, Ph.D.), location of the pharmacy, total experience, and type of outlet. To determine the knowledge of the participants, 9 questions were asked in the second section using three answer options; “yes”, “no”, and “unsure”. These questions were asked per CP qualifications. Three examples were posed in which either the given case was appropriate (yes) or not as per CP practice (no). The third section focused on the attitude of CPs towards nonprescription antibiotics, with a total of 8 questions asked. 

A 5-point Likert scale (strongly agree, agree, neutral/uncertain, disagree, and strongly disagree) was used. The first question focused on the fact that “ABR/AMR is an important public health issue and resistance is mainly developed due to the misuse of antibiotics”. The last section of the questionnaire was about the practices of CPs regarding antibiotic dispensing, counseling, and educating patients on antibiotic resistance. A similar Likert scale was also used in section four from “strongly agree” to “strongly disagree”. The above instrument’s reliability was confirmed by using Cronbach α, where a value of more than 7 was considered an acceptable range indicating a good fit for internal consistency. Additionally, the scores for knowledge of the participants were considered by assigning one point to each correct item, either “yes” or “no”.

#### 2.2.3. Data Collection

An online Google form of the questionnaire was developed with several sections, where the objectives of the study were mentioned on the first page. Monographs of antibiotics that should be used with care were mentioned on every page. Depending on the participants’ preference and convenience, different social media platforms including “WhatsApp” and “Facebook messenger” were used to administer the survey. Subsequently, the full and functional questionnaire was sent to the CPs across the country. To increase participation, the participants were allowed to answer all of the questions by just clicking their answers on a given link. 

The snowball and convenient sampling methods were used for data collection. The participants were requested to share the questionnaire with their CP colleagues in the locality. Pharmacists within the age group of 24–28 years or more, working as a CP (full-time, part-time, or linked with community pharmacy), and living in Pakistan were included in this study. All data were collected in the form of responses through the Google form (https://docs.google.com/forms/), which was subsequently transformed into a data analysis software. Those who completed the first part of the study were invited for the second part via telephonic interviews or by using choice applications convenient to the participant. 

#### 2.2.4. Data Analysis 

The participants’ responses were collected and transferred to SPSS (Statistical Package for Social Sciences, Chicago, IL, USA, Version 22), where different statistical techniques were employed based on the data type. For the frequencies and percentages, descriptive statistics were used and were presented in tables. The median difference in the knowledge, attitude, and practices scores were determined through the Kruskal–Wallis and Mann–Whitney statistical tests and presented in table with median and IQR (Interquartile range defines the middle 50% of values from “lowest to highest” when ordered) A *p*-value < 0.05 was considered statistically significant.

### 2.3. Phase 2: Qualitative Study

#### 2.3.1. Study Instrument (Interview Guide) 

For the qualitative part, an interview guide was developed based on qualitative and explorative study designs [39] to gauge the knowledge gap in a CP’s views on antibiotics misuse and how to curb the use of nonprescription antibiotics along with ABR matter. Semi-structured interviews are useful tools, especially for exploratory studies. They have many advantages such as the ability and flexibility to yield an in-depth investigation of knowledge, experiences, and intention of the participants towards a particular issue [40,41]. Pilot interviews were undertaken for the trial to confirm the protocol’s clarity and validity. The interview scheme contained themes, subthemes, and questions (a total of 16 questions) with subsections (Supplementary File S1).

#### 2.3.2. Data Collection (Interviews)

The participants were invited for qualitative interviews through WhatsApp voice calls following their informed consent. In-depth online interviews were conducted with the CPs to determine their overall perceptions about the rational use of antibiotics and antibiotic resistance. Participants were allowed to answer in English or Urdu (national language) per their preference. Each interview lasted for about 20–40 min. A voice recorder was used with consent for the recording, which was stored for further analysis.

#### 2.3.3. Analysis

General inductive thematic analysis was performed on the data and was concluded with developing themes [42,43]. All recorded interviews were carefully played back, and observations were cautiously noted. Subsequently, the interviews were transcribed verbatim from the audio recordings. The major themes and subthemes were finalized, and codes were assigned to the meaningful data. The point at which no other themes and concepts are developed is known as the saturation point [39,44]. Therefore, the interviews were stopped after the 17th interview when the saturation level was reached. Finally, for confirmation of saturation, another four interviews were conducted. 

## 3. Results

### 3.1. Phase 1: Quantitative

#### 3.1.1. Demographics

A total of 180 participants completed the survey, with males being the majority (64.4%). Most (64.4%) participants were aged 29 to 33 years, held at least a bachelor’s degree in pharmacy (77.2%), and were from Punjab (29.4%). Nearly one-half of the participants (46.1%) had 1 to 2 years of experience in community pharmacy practices (Table 1).

#### 3.1.2. Knowledge of Antibiotics and Antibiotic Resistance 

Generally, the participants had good knowledge of certain aspects of antibiotic use including of multidrug-resistant organisms that are responsible for ABR (65.6%, median = 1, and IQR = 1). The participants were also able to identify that the ABR rate is higher in hospital settings in Pakistan (71.7%, median = 1, and IQR = 1). Similarly, a vast majority of the participants were aware of penicillin cross-sensitivity (60.0%, median = 1, and IQR = 1). However, knowledge in differentiating some groups of ABR patterns may be slightly deficient (31.1%, median = 1, and IQR = 1) (Table 2).

#### 3.1.3. Attitude about Antibiotics

Generally, the attitude of most participants was positive towards antibiotics. About 90.0% of participants identified ABR as a critical issue of public health (median = 1 and IQR = 0). A large number of participants either “agreed” (37.8%) or “strongly agreed” (30.0%) with the statement that the discovery of newer antibiotics will solve the issue of ABR (median = 2 and IQR = 2). Moreover, the participants believed that counseling should be provided to the patients during dispensing of antibiotics to improve their compliance with drug therapy (69.4%, median = 1, and IQR = 1). A large number of participants considered that dispensing antibiotics without a prescription is a severe issue (68.3%, median = 1, and IQR = 1). Most participants agreed with the statement that “Antibiotics are sometimes prescribed without medical prescription because patient neither have the time nor the money to see a physician” (43.9%, median = 2, and IQR = 1) (Table 3). 

#### 3.1.4. Practices about Antibiotics

Overall, antibiotic practices among the CPs were reasonable. For example, more than half of the participants indicated that they educated their patients on the rational use of antibiotics (52.8%, median = 1, and IQR = 1). Approximately 47.2% indicated that they partook in campaigns focused on optimal antibiotic use (median = 2 and IQR = 1). Likewise, 48.3% informed that they indulged in practices that prevent infection transmission within the community (median = 2 and IQR = 1). A large number of participants agreed that they work in collaboration with other healthcare professionals to limit infection and to improve ASP activities (50.0%, median = 1, and IQR = 0.75). Approximately 41.1% of participants agreed with the statement “sometimes screen the antibiotics following local guidelines before dispensing” (median = 1 and IQR = 1.75). However, only 18.9% of participants agreed that they dispense antibiotics without a prescription (median = 4 and IQR = 1) (Table 4). 

There was a significant association between gender and experience with knowledge on antibiotics (Table 5). Knowledge on antibiotics tends to be higher among female participants (median knowledge score = 2 and IQR = 1 vs. median knowledge score = 1 and IQR = 0). Likewise, participants with at least 1–2 years of working experience had more knowledge on antibiotics than other age groups (median knowledge score = 2 and IQR = 1 vs. median knowledge score = 1 and IQR = 1). 

#### 3.1.5. Phase 2: Qualitative Study

Overall, 21 in-depth online interviews were conducted with a mean duration of 25 min. There were more males (*n* = 14) compared with females. The mean age was between 26 and 40 years, with five CPs having master’s degrees in pharmacy. An analysis of the data produced six themes, 10 subthemes, and 25 categories (Table 6).

#### 3.1.6. Theme I: Common Infectious Diseases

The most common types of infections and misconceptions about antibiotics use in patients: respiratory and urinary tract infections are the most commonly reported. The majority of the CPs agreed with the above that infections are most commonly reported, but two CPs had some experienced with typhoid patients.

“No doubt respiratory tract infections are common but, in our locality, the majority of prescriptions were filled for typhoid patients daily.”

(CP: 3)

“Skin infections are very common in the locality.”

(CP: 19)

#### 3.1.7. Theme II: Misuse (Self-Medications) of Antibiotics

How to reduce self-medication with antibiotics and CPs, the physician’s role, and how to improve the appropriate use of antibiotics: consumers do not know what antibiotics are, and when and how to use them appropriately.

“The majority of patients use antibiotics without pharmacist consultation; even when they were counseled, they sometimes do turn up for a refill of antibiotic prescription.”

(CP: 2)

“Most consumers do not know that metronidazole is an antibiotic and always demands its use, although they may not need it. Among pediatrics, the most commonly prescribed antibiotics are amoxicillin with clavulanic acid, cefixime and azithromycin for which physicians are sometimes called for consultation.”

(CP: 3)

Most respondents believed that pharmacists and physician linkages for consultation on prescribed antibiotics are important in curbing the issue of antibiotic resistance.

“Unfortunately, throughout the country, CPs have no such easy access to physicians as per job experience at a community pharmacy.”

(CP: 10)

### 3.2. Theme III: Patient Counseling and Education 

CP services that can be offered to enhance the rational use of antibiotics:

“We (pharmacy staff) are always trying to do counseling on when, how, antibiotics are to be used and their adverse events.”

(CP: 5)

“Patients must understand both viral and bacterial diseases; I always discourage antibiotic prescriptions for flu and the common cold.”

(CP: 8)

All CPs are urged to spend more time on patient counseling and education.

“If a patient receives an antibiotic without pharmacist’s consultation, there pose a risk for medication error. A patient has merely utilized three doses and left the rest of the antibiotics prescribed without taking them.”

(CP: 15)

“Educate your patient, this is the least CPs can do in a short time.”

(CP: 4)

#### 3.2.1. Theme IV: Awareness Campaigns at the Community Level

Awareness campaigns on antibiotic resistance in order to enhance public knowledge on using antibiotics with care: “Awareness is the key to curbing antibiotic resistance in Pakistan where the majority of the population are unaware on antibiotic resistance.” (CP: 18)

“Booklets and pamphlet which comprised of graphics to indicate antibiotics misuse and their consequences should be distributed along with antibiotic prescriptions.”

(CP: 9)

#### 3.2.2. Theme V: Training Programs for CPs 

Short- or long-term training for CPs to assure the AMS at the community level and to provide an update on the current situation of ABR: “Training is the most important area for CPs where all hospitals/community pharmacies across the country should have at least a training session once a month.” (CP: 5).

“Hands-on training, seminars and other activities will have a positive impact on curbing the ABR issue where every CP should have current knowledge on ABR.”

(CP: 6)

#### 3.2.3. Theme VI: Factors Leading to Nonprescription Antibiotics

Budget and time constraints, incompetent staff, the absence of CPs, and a lack of law enforcement or regulations: “Nonprescription antibiotics are a multifactorial issue especially in the rural areas where qualified CPs are hardly available.”(CP: 7)

“The absence of CPs at pharmacies/medical stores are the actual delinquent in our society where a strict check and balance system should be implemented.”

(CP: 9)

“Most pharmacies have incompetent staff who dispensed antibiotics without prescription.”

(CP: 13)

#### 3.2.4. Theme VII: CP-Based Interventions

Unavailability of the prescriber/physician at call and consultation time: “Pharmacists tend to wish for physician’s contact and conduct interventions especially for duplicate antibiotics including prescriptions although CPs tend to face the issue of unavailability of the physician on call.”(CP: 16).

“Pakistani laws do not allow a pharmacist to prescribe or make changes without consultation with their prescriber; however, when recommended doses have been exceeded, CPs change the dose frequency accordingly.”

(CPs: 19)

#### 3.2.5. Theme VIII: How to Curb Antibiotic Resistance at the Level of Community Pharmacy

How to engage healthcare professionals to combat ABR: “Community pharmacies are the first gate for the provision of medication; healthcare providers should engage pharmacies to ensure prescription accuracy.”(CP: 5).

“Scientific seminars can highlight ABR issues affected around the world; they are platforms for interaction with healthcare providers in which collective efforts can help reduce ABR.”

(CP: 15)

#### 3.2.6. Theme IX: Strategies to Overcome Barriers

Suggestions related to the physician, government, and CP for improving antibiotic use in the community: “The main barrier is the communication gap between pharmacists and physicians. The gap should be reduced for effective and collaborative work.”(CP: 5)

“Pharmacists must be present at the pharmacy during their duties; the presence of CPs increases the importance of a pharmacist in the community.”

(CP:18)

## 4. Discussion

In most developing countries such as Pakistan, the sale of antimicrobials in community medicine drug outlets are unregulated, without the contribution of a licensed pharmacist, and dispensed antibiotics are often without a valid medical prescription [45]. To the best of our knowledge, this is the first study to access the knowledge, attitude, and practice on antibiotics and its resistance among community pharmacists in Pakistan which can help promote rational drug use locally. Therefore, there remains a large gap that can still be filled to improve the therapeutic potentials of antibiotics while keeping side effects low. 

In the present study, the majority of CPs understand antibiotics and antibiotic resistance. However, their knowledge of the mechanism of resistance in a different group of antibiotics remains lacking, as previously reported [46,47,48,49]. Overall, CPs have good knowledge about antibiotics that can cause allergic reactions, also previously reported [50]. Sufficient knowledge related to antibiotic use may avert irrational dispensing of antibiotics and may help decrease antibiotic resistance [51,52]. In our study, the majority of the CPs strongly agreed that antibiotic resistance usually occurs when there is misuse and overuse of antibacterial agents, with antibiotic resistance being one of the major public health problems in Pakistan [46,47,49,53].

In our study, the attitudes of most CPs tended to be positive towards antibiotics and ABR. It is encouraging to see that most CPs tend to dispense antibiotics only on prescription, also previously reported [49,54,55]. As reported by the World Health Organization (WHO) [56], the majority of CPs strongly agreed that the discovery of new antibiotics may solve the issue of ABR.

Counseling should be provided to patients during dispensing of antibiotics to improve their adherence, as also recommended in other studies [57,58]. Antibiotic dispensing without a medical prescription is one of the critical issues in Pakistan, especially in the capital and Punjab province [59,60], which are highly populated. A positive attitude towards ABR will make CPs more confident when dispensing antibiotics. Some negative attitudes may influence CPs to dispense antibiotics without a prescription [61]. 

Good practices have a long-lasting effect, and in our finding, in-practice CPs have reasonable knowledge towards antibiotics and ABR, with approximately 47.2% of the participants focused on optimal antibiotic dispensing, as also previously reported [62,63]. Malpractices towards ABR call for a swift educational intervention programs amongst CPs, while the absence of continuing education and other confounding factors can affect practices [64]. When dispensing antibiotics, guidelines must be followed. Nevertheless, the majority of CPs agreed that, sometimes, they tend to screen antibiotics with local guidelines, as also similarly reported by Sarwar et al., from Punjab Pakistan, and by Costa et al., from Europe [49,65], indicating that it is important that the local guideline aligns with the accepted standards.

Alarmingly, our study indicated that the majority of CPs hardly collaborated with other healthcare professionals in ABR and ASPs. Similarly, Hayat et al. and Khan reported the same [66,67], indicating that there is room for interprofessional education. Educational interventions must therefore be put in place to improve the rational dispensing and prescribing of antibiotics, which can help reduce ABR [68]. 

The present study indicated that the majority of the CPs agreed that suitable training, workshops, and other relevant events on ABR are recommended. Training for CPs can help encourage the appropriate dispensing and use of antibiotics, thus helping to curb ABR [58]. There are many reasons for misuse and self-medication with antibiotics in Pakistan, where prescription-only antibiotics play a role in self-medication. Due to this fact, patient counseling for antibiotics is an important area for CPs, on top of patient education [69]. One of the important issues is awareness of antibiotic misuse, for which an antibiotics week was celebrated in October all around Pakistan to curb inappropriate antibiotics use [67].

ABR is not only an issue in LMICs but also in developed countries, making it a global issue [67]. Although our findings may represent that in LMICs, it is not very different from that of Poland, UK, Germany, Sweden, Italy, Lithuania, Cyprus, Siberia, and Hong Kong [67]. Evidence on operative and achievable stewardship interventions in LMICs remains inadequate with numerous challenges for interventions. Nevertheless, several initiatives at the global and local levels in Latin America, Asia, and Africa have revealed that effective interventions are possible in LMICs, although contextualization is important [70]. Moreover, all healthcare systems must develop initiatives and solutions to reduce antibiotic consumption and antimicrobial resistance [71,72]. Therefore, strengthening and enhancing the CPs’ role in developing nations have the potential to positively affect ABR globally [73,74].

Strict implementation of regulations is the need of the hour, especially in Pakistan, where many pharmacies still run without a qualified pharmacist [30]. All these factors contribute to global ABR prevalence enormously if timely actions are not taken. Therefore, the current mixed-methods study highlights key issues related to antibiotics and ABR along with the knowledge, attitude, and practices of CPs regarding antibiotics. The rational use of antibiotics can be improved by discouraging self-medication, by enhancing patient education and awareness, as well as by strengthening the already defined strategies to be implemented. 

Nevertheless, our study is not without limitations. First, both full- and part-time were recruited since there is a lack of full-time CPs in Pakistan. Secondly, the two-phase study poses the possibility of a recall bias. Third, a convenience sampling technique was used. Future studies should address these issues to have a better representation of the research question. 

## 5. Conclusions

Overall, CPs in Pakistan had good knowledge and a positive attitude but poor to moderate practices towards antibiotics and ABR. Nevertheless, there is an urgent need for training, awareness campaigns, patient education, and educational interventions at the community level with ASPs strictly practiced. The government should implement the laws and ensure that only qualified pharmacists are allowed to practice to stem the issue with ABR. 

## Figures and Tables

**Table 1:** Characteristics of respondents (*n* = 180)**.**

Variable	*N* (%)
Gender	
Male	116 (64.4%)
Female	64 (35.6%)
Age (years)	
24–28	47 (26.1%)
29–33	116 (64.4%)
>33 *	17 (9.4%)
Education	
B Pharmacy/Pharm D	139 (77.2%)
Postgraduation (MPhil/PhD)	41 (22.8%)
Region	
* KPK	36 (20.0%)
Sindh	37(20.6%)
Punjab	53 (29.4%)
Baluchistan	11 (6.1%)
Islamabad	23 (12.8%)
* AJK	10 (5.6%)
* GB	10 (5.6%)
Experience (years)	
Less than one year (<1)	43 (23.9%)
More than one (>1 and <2)	83 (46.1%)
More than two (>2) or more	54 (30.0%)

* KPK: Khyber Pakhtunkhwa, AJK: Azad Jammu and Kashmir, GB: Gilgit Baltistan; * >33: more than 33 and less than 60 years.

**Table 2:** Knowledge of respondents about antibiotics.

Questions	Yes (%)	No (%)	Unsure (%)	Median (IQR)
‘Multidrug resistant organisms’ are responsible for ABR.	118 (65.6)	18 (10.0)	44 (24.4)	1 (1)
Resistance (DNA) in bacteria can be transferred to other bacteria through a virus carrier.	99 (55.0)	39 (21.7)	42 (23.3)	1 (1)
Is it true that AMR is higher in hospital settings than in community settings?	129 (71.7)	40 (22.2)	11 (6.1)	1 (1)
AMS aims to achieve an effective clinical outcome with less toxicity and ADRs	149 (82.8)	19 (10.6)	12 (6.7)	1 (0)
Penicillin, cephalosporin, and fluoroquinolone are β-lactam antibiotics. Beta-lactamase producing bacteria should be considered.	112 (62.2)	56 (31.1)	12 (6.7)	1 (1)
Amoxicillin-allergic patients (anaphylaxis type) should not use Cephalexin.	108 (60.0)	42 (23.3)	30 (16.7)	1 (1)
Is the statement correct? A pharmacist should dispense amoxicillin 1500 mg a day, 7 days for a 24-year-old patient with allergic rhinitis, a high-grade fever, rhinorrhea, sore throat, and no known drug allergies.	48 (26.7)	92 (51.1)	40 (22.2)	2 (1)
Is the statement correct? A pharmacist should dispense only mineral powder in case of a 1.8-year-old baby girl with watery diarrhea, no mucous/bloody stool, no fever, no vomiting, and no known drug allergies.	93 (51.7)	47 (26.1)	40 (22.2)	1 (1)
Is the statement correct? A pharmacist should dispense dicloxacillin 250 mg four times a day for 5 days to prevent infection in case of a 26-year-old female who has a skin abrasion wound on her right arm without exudates for 2 days, that was limited to the subcutaneous layer, with mild tenderness, no swelling, no active bleeding, no fever, and no known drug allergy.	71 (39.4)	50 (27.8)	59 (32.8)	2 (2)

AMR = Antimicrobial resistance, AMS = Antimicrobial stewardship program, ADR = Adverse drug reaction.

**Table 3:** Attitude of respondents about antimicrobial stewardship programs (ASPs).

Questions	SA (%)	A (%)	U/N (%)	DA (%)	SD (%)	Median (IQR)
	Agree			Disagree		
Is AMR being an important public health issue?	162 (90.0)	16 (8.9)	2 (1.1)	0 (0.0)	0 (0.0)	1 (0)
A patient who takes antibiotics has higher risk of developing resistance.	91 (50.6)	61 (33.9)	8 (4.4)	20 (11.1)	0 (0.0)	1 (1)
New antimicrobials/antibiotics discoveries and development can solve the AMR problem.	55 (30.6)	68 (37.8)	22 (12.2)	32 (17.8)	3 (1.7)	2 (2)
The use of antibiotics in livestock animals is an important cause of the appearance of new resistance to pathogenic agents in humans.	46 (25.6)	77 (42.8)	53 (29.4)	4 (2.2)	0 (0.0)	2 (2)
In all cases where antibiotics are dispensed, patients must be advised about complying with the treatment.	125 (69.4)	45 (25.0)	3 (1.7)	5 (2.8)	2 (1.1)	1 (1)
Antibiotics are sometimes dispensed without a medical prescription because the patient is known to have difficulty in obtaining a medical consultation.	53 (29.4)	74 (41.1)	26 (14.4)	14 (7.8)	13 (7.2)	2 (2)
Antibiotics are sometimes prescribed without medical prescription because the patient is known to have neither the time nor the money to see a physician.	66 (36.7)	79 (43.9)	14 (7.8)	14 (7.8)	7 (3.9)	2 (1)
Dispensing antibiotics without prescription is a serious issue.	123 (68.3)	46 (25.6)	10 (5.6)	1 (0.6)	0 (0.0)	1 (1)

SD = Strongly disagree, D = Disagree, U/N = uncertain, A = Agree, SA = Strongly agree, ASP = Antimicrobial stewardship program, IQR = Interquartile range.

**Table 4:** Participants’ responses to ASPs.

Questions	SA (%)	A (%)	N/U (%)	DA (%)	SD (%)	Median (IQR)
	Agree			Disagree		
I educate patients on the use of antimicrobials and resistance-related issues.	95 (52.8)	69 (38.3)	7 (3.9)	9 (5.0)	0 (0.0)	1 (1.00)
I partake in antimicrobial awareness campaigns to promote the optimal use of antibiotics.	63 (35.0)	85 (47.2)	23 (12.8)	9 (5.0)	0 (0.0)	2 (1.00)
I have some lack of knowledge on continuing education in antimicrobial use and resistance topics.	36 (20.0)	64 (35.6)	23 (12.8)	51 (28.3)	6 (3.3)	2 (2.00)
I make efforts to prevent or reduce the transmission of infections within the community.	64 (35.6)	87 (48.3)	28 (15.6)	1 (0.6)	0 (0.0)	2 (1.00)
I collaborate with other health professionals for infection control and antimicrobial stewardship.	45 (25.0)	91 (50.6)	30 (16.7)	11 (6.1)	3 (1.7)	1 (0.75)
I ask the patient’s history and symptoms of their infections before deciding to dispense antimicrobials.	80 (44.4)	66 (36.7)	24 (13.3)	9 (5.0)	1 (0.6)	2 (1.00)
I sought additional clinical information (e.g., drug interaction, ADRs & allergy) before deciding to dispense the antibiotics.	63 (35.0)	88 (48.9)	19 (10.6)	9 (5.0)	1 (0.6)	2 (1.00)
I screen the antibiotics following local guidelines before dispensing.	45 (25.0)	74 (41.1)	42 (23.3)	15 (8.3)	4 (2.2)	1 (1.75)
I dispense antibiotics with complete clinical information (e.g., drug interaction, ADEs and allergy) given.	51 (28.3)	93 (51.7)	24 (13.3)	10 (5.6)	2 (1.1)	2 (1.00)
I dispense antibiotics without a prescription.	8 (4.4)	34 (18.9)	32 (17.8)	65 (36.1)	41 (22.8)	4 (1.00)

ADEs = Adverse drug events.

**Table 5:** Median score of knowledge about antibiotics, and attitudes of and practice in ASPs.

Variables	Knowledge Score Median (IQR)	*p*-Value	Perception Score Median (IQR)	*p*-Value	Practice Score Median (IQR)	*p*-Value
Gender *						
Male	1.00 (0.00)		1.50 (1.00)		2.00 (1.00)	
Female	2.00 (1.00)	<0.001	1.50 (1.00)	0.914	2.00 (0.50)	0.382
Age (years) **						
24 to 28	1.00 (1.00)	0.273	2.00 (1.00)	0.465	2.00 (1.00)	0.451
29 to 33	1.00 (1.00)		1.50 (1.00)		2.00 (0.50)	
>33	1.00 (0.00)		1.50 (1.00)		2.00 (1.00)	
Education *						
B Pharmacy or PharmD.	1.00 (1.00)	0.324	1.50 (1.00)	0.785	2.00 (1.00)	0.045
M.Phil./PhD.	1.00 (1.00)		1.50 (1.00)		1.50 (0.00)	
Regions **						
KPK	1.00 (0.75)	0.148	1.50 (1.00)	0.366	2.00 (0.88)	0.573
Sindh	1.00 (1.00)		1.50 (1.00)		2.00 (0.00)	
Punjab	1.00 (1.00)		2.00 (1.00)		2.00 (1.00)	
Balochistan	2.00 (1.00)		1.50 (1.00)		2.00 (1.00)	
Islamabad	1.00 (1.00)		1.50 (1.00)		2.00 (1.00)	
AJK	1.50 (1.00)		1.00 (0.63)		2.00 (1.00)	
GB	2.00 (2.00)		2.00 (1.00)		2.00 (0.88)	
Experience (years) **						
<1	1.00 (1.00)		2.00 (1.00)		2.00 (1.00)	
1 to 2	2.00 (1.00)	<0.001	1.50 (1.00)	0.285	2.00 (1.00)	0.176
>2	1.00 (0.00)		1.50 (1.00)		2.00 (0.50)	
Type of outlet *						
Pharmacy	1.00 (1.00)	0.099	1.50 (1.00)	0.260	2.00 (1.00)	0.794
Other	1.00 (0.00)		2.00 (1.00)		2.00 (0.75)	

* Mann–Whitney Test, ** Kruskal–Wallis Test.

**Table 6:** Details on the themes, subthemes, and categories.

Theme	Subtheme	Categories
Infections	The most common type of infections in the community	Respiratory tract infections (upper and lower respiratory tract infections) are the most prevalent.Urinary tract infections are also common.Other infections
Misuse of antibiotics	Self-medication with antibiotics	Patients usually do not consult with a medical practitioner.People have their suggestions for treatment with antibiotics.Patients mostly do not complete the full course of antibiotics.
	Access to antibiotics	Easy access to antibiotics especially in rural areas.The absence of a qualified person (pharmacist) at pharmacy/drug outlets.
	Physician–pharmacist linkage and coordination	Physicians/prescribers and pharmacist linkages are not unavailable. It is sometimes hard to understand the prescriptions including antibiotics. Patients always rely on and demand what a physician has prescribed. Consultation with a physician for patients’ medications is very rare in Pakistan.
Patient education	Counseling	Sometimes, patients do not listen to the CP who tries to counsel the patient on the prescribed antibiotics.Some modern pharmacies set up in some big cities have enough space and pharmacists for counseling.
	Awareness	Awareness is the key to curbing antibiotic misuse.Awareness campaigns, camps, seminars, and awareness walks would be helpful.Written charts and brochures in local languages could indicate the importance of antibiotics.
Nonprescription antibiotics	Antibiotics without prescription	It is a very important and common issue particularly in the rural areas.Sometimes, patients bring empty antibiotic strips, bottles, or leaflets demanding to be given similar antibiotics. The pharmacist will not dispense antibiotics without a prescription. However, off duty, the staff may not hesitate to dispense.
Antibiotic resistance	The rising issue in Pakistan	Almost all participants have some knowledge on antibiotic resistance.Most CPs believed that patients should stop the antibiotics after 3–4 doses after they felt well.The pharmacists have pledged to play their roles as CPs more than before.
Suggestions for prescription-only antibiotics	Physicians/medical practitioner role	The physician/prescriber should prescribe antibiotics for the patient based on the situations, and over-prescribing must be avoided, which leads to a cutoff in the doses of the antibiotic for the one who cannot afford it.
	CPs role	Pharmacies/drug outlets must have pharmacists. Awareness of CPs and their roles in the healthcare system should be explored at the community level.CPs should not dispense the antibiotics without an authentic prescription, and the records should be maintained.
	Government and legislation role	The healthcare system must be improved as three provinces are still deprived of such legislation.The number of healthcare professionals must be increased so the prescribers would have more time for patient consultations.Outdated pharmacy and drug laws must be renewed with modern regulations in Pakistan.

## Supplementary Materials

The following are available online at https://www.mdpi.com/1660-4601/18/3/1320/s1, S1: Questionnaire and interview guide.
