# Supplementary material for: Knowledge, Attitude, and Practice on Antibiotics and Its Resistance: A Two-Phase Mixed-Methods Online Study among Pakistani Community Pharmacists to Promote Rational Antibiotic Use
Source: Int J Environ Res Public Health. 2021 Feb 1;18(3):1320. doi: 10.3390/ijerph18031320 (PMC7908617; doi:10.3390/ijerph18031320)
Supplement: Supplementary file 1 [file ijerph-18-01320-s001.pdf]

Participant Information Pack

Content:

Participant Information Sheet
Informed Consent
Informed Consent declaration
Questionnaire (Quantitative)
Questionnaire (Interview Guide)

A. PARTICIPANT INFORMATION SHEET

You are being invited to participate in a research study titled *“Knowledge, Attitude, and Practice on Antibiotics and Its Resistance: A Two-Phase Mixed-Methods Online Study among Pakistani Community Pharmacists to Promote Rational Antibiotic Use”.*

Purpose of study:

This study aims to assess pharmacists' knowledge, attitude, and practice regarding antimicrobial use over the counter and antibiotic resistance in Pakistan and will take you approximately 10-15minutes to complete. After completion of the first part upon your willingness, you can participate in the second part of the study for which we will call you through WhatsApp or other applications feasible to you.

Your Participation:

Your participation in this study is entirely voluntary and you can withdraw at any time. We believe there are no known risks associated with this research study; however, as with any online related activity, the risk of a breach is always possible. To the best of our ability, your answers in this study will remain confidential. We assure you that all data collected will be anonymous. All data will be stored electronically on password-protected media for a maximum of three years. All data collection, storage, and processing will comply with the principles of the Data Protection.

B. INFORMED CONSENT

You will be asked to sign an informed consent stating that you understand the nature of the study and what is required from you in the study. Participation in this study is voluntary and will not affect your privacy. You are free at any time to change your mind and withdraw from the study without needing to justify your decision.

* By completing this survey please click AGREE to indicate that:

- You are a pharmacist aged 18 years or older
- You practice as full-time or part-time in a community pharmacy in Pakistan
- You have read the participant information sheet associated with this study

Sign (optional) ____________

PART-I

Questionnaire

Section 1: Demographics

Please tick the relevant box or write your answer when appropriate

Gender		
Male		
Female		
Age (years)		
19 to 26		
27 to 34		
>35		
Education*		
B Pharmacy/Pharm D		
M.Phil.		
Region		
KPK		
Sindh		
Punjab		
Balochistan		
Islamabad		
AJK		
GB		
Experience (years)		
< 1		
1 to 2		
>2		
Type of drug outlet		
Pharmacy Type		

Section 2: Knowledge

Questions	Yes (%)	No (%)	Unsure (%)
The 'Superbugs" are responsible for antibiotic resistance, including (bacteria, fungal, parasites, and viruses).			
Resistance (DNA) in bacteria can transfer to other bacteria through a virus carrier.			
is it true that AMR in a hospital setting is higher than community setting?			
AMS aims to achieve an effective clinical outcome with less toxicity and ADRs			
Penicillin, cephalosporin, and fluoroquinolone are β-lactam antibiotics, It needs to consider Beta-lactamase producing bacterial.			
Amoxicillin allergic patients (Anaphylaxis type) should not use Cephalexin.			
is it appropriate? When a pharmacist dispenses amoxicillin 1,500 mg a day, 7 days for a 26-year-old male with allergic rhinitis, has high-grade fever rhinorrhea, and sore throat, and no known drug allergies.			
Is it appropriate? When a pharmacist dispenses only mineral powder in case of a 2year-old boy with watery diarrhea, no mucous/bloody stool, no fever, no vomiting, no known drug allergies.			
Is it appropriate? When a pharmacist dispenses dicloxacillin 250 mg four times a day for 5 days to prevent infection in case of a 24-year old male who has had a skin abrasion wound on his right arm without exudates for 2 days, limited to the subcutaneous layer, m			

Section 2: Attitude

Questions	SA (%)	A (%)	N (%)	DA (%)	SD (%)
AMR is an important public health issue?					
The fact that a patient is taking antibiotic increases the risk of developing resistance.					
New antimicrobials discoveries and development can solve the AMR problem.					
The use of antimicrobials in livestock animals is an important cause of the appearance of new resistance to pathogenic agents in humans.					
In all cases where antibiotics are dispensed, patients must be advised about complying with the treatment.					
Antibiotics are sometimes dispensed without medical prescription because the patient is known to have difficulty in obtaining a medical consultation.					
Antimicrobials are sometimes prescribed without medical prescription because the patient is known to have neither the time nor the money to see a physician					
Dispensing antibiotics without prescription is a serious issue.					

Section 3: Practices

Questions	SA (%)	A (%)	N (%)	DA (%)	SD (%)
I educate patients on the use of antimicrobials and resistance-related issues.					
I take part in antibiotics awareness campaigns to promote the optimal use of antimicrobials.					
I have lack to continuing education in antibiotics use and resistance topics.					
I make efforts to prevent or reduce the transmission of infections with in the community					
I collaborate with other health professionals for infection control and antimicrobial stewardship.					
I ask patient’s history and symptom of their infections before deciding to dispense antibiotics.					
I sought additional clinical information (e.g. drug interaction, ADRs, allergy, etc.) before deciding to dispense the antibiotics.					
I screen the antibiotics in accordance with local guidelines before dispensing.					
I dispense antibiotics with complete clinical information (e.g. drug interaction, ADRs, allergy, etc.)					
I dispense antibiotics without a prescription.					

PART-II

Interview Guide

COMMUNITY PHARMACIST INTERVIEW GUIDE

II. How to improve the rational use of antibiotics

1.Demogrsaphics:

Name: ____

Age: ____

Gender: ____

Place: ____

Degree: ____

Current Job: ____

Name of Pharmacy: ____

Total Years of Experience: ____________________________________

Community pharmacist interview Guide

1. In your practice, what are the most common types of infectious diseases you encounter?

Prob: what are the most common infections common in your community?

2. in your opinion, what are some common misconceptions of patients about antibiotic use?

Probe: self-medication, use of antibiotics in viral diseases such as flu, stoppage of antibiotics without consultation with a doctor, side effects of antibiotics.

3. Which strategies should be considered the highest priority to resolve from the perspective of healthcare providers (Physician-Pharmacist linkage)?

4. What are some actions you currently use to improve antibiotic use in your area of practice?

Probe: education and counseling

5. In your opinion, what are some services that community pharmacists could offer to enhance the appropriate use of antibiotics by the general population in the community?

Probe: awareness campaigns, public education

6. Do you feel community pharmacists need additional training to implement suggested strategies?

Probe: short-term training or long-term training

7. What are some barriers to implementing interventions you have indicated to improve antibiotic use at your practice site?

Probe: budget constraints, time constraints, incompetent staff, absence of community pharmacists, lack of enforcement of laws to restrict antibiotic prescribing without a prescription.

8. What are some strategies to overcome barriers which would allow community pharmacists to provide services relating to improving antibiotic use at your practice site?

9. Is there anything else you would like to say about improving antibiotic use in the community through pharmacist lead interventions?

10. Others__________________
11.

Summary of the Interview Guide	
Infections	Most common type of infections in the community
Misuse of antibiotics	Self-medication with antibiotics
	Access to antibiotics
Patient education	Counselling
	Awareness
Nonprescription antibiotics	Antibiotics without prescription
Antibiotic resistance	Rising issue in Pakistan (Suggestions)
